# Three-dimensional assessment of macromastia: linking breast morphology to quality of life

**DOI:** 10.3389/fgwh.2026.1822760

**Published:** 2026-07-01

**Authors:** Huijing Wang, Xian Jin, Xinghong Chen, Xingyu Shi, Jing Wang, Chenggang Yi

**Affiliations:** Department of Plastic Surgery, The Second Affiliated Hospital, Zhejiang University School of Medicine, Hangzhou, Zhejiang, China

**Keywords:** breast volume, BREAST-Q, gigantomastia, macromastia, quality of life, three-dimensional imaging

## Abstract

**Background:**

Macromastia is associated with substantial physical and psychosocial burden, yet existing diagnostic thresholds, particularly for gigantomastia, are largely derived from weight-based criteria established in Western populations. These standards may be suboptimal for preoperative assessment in Asian women, where breast composition and body habitus differ.

**Methods:**

In this cross-sectional study, adult women with breast volumes > 400 mL were recruited from a tertiary plastic surgery center. Preoperative breast morphology was assessed using the VECTRA® XT 3D imaging system, including breast volume, breast width, SNN distance, and ptosis grade. QoL was evaluated using the BREAST-Q Reduction/Mastopexy Module (version 2.0). Associations among morphological parameters, BMI and BREAST-Q domains were comprehensively analyzed.

**Results:**

A total of 145 patients underwent 3D imaging and 168 completed the BREAST-Q questionnaire. Fifty-three patients completed both assessments and were included in analyses integrating breast morphology and patient-reported outcomes. Breast volume was positively correlated with BMI and ptosis severity (*P* < 0.05). SNN distance demonstrated a stronger correlation with ptosis grade (*ρ* = 0.657, *p* < 0.001). Patients with breast volumes ≥ 1,000 mL demonstrated significantly greater ptosis severity and lower physical well-being scores compared with those with moderate macromastia. BMI showed stronger unadjusted associations with psychosocial well-being, sexual well-being, and satisfaction with breasts than breast volume alone. However, these associations were attenuated after multivariable adjustment.

**Conclusions:**

Three-dimensional imaging combined with BREAST-Q assessment provides complementary information on the anatomical and patient-reported burden of macromastia. Breast volumes of 1,000 mL or greater were associated with increased ptosis severity and poorer physical well-being and may represent a clinically relevant threshold warranting further investigation. These findings support a multidimensional approach to preoperative evaluation that incorporates breast morphology, body habitus, and patient-reported outcomes.

## 1 Introduction

Breast hypertrophy, also referred to as macromastia, is characterized by excessive breast tissue growth beyond normal physiological limits. This condition is frequently associated with substantial physical and psychological morbidity, including musculoskeletal pain, postural alterations, limitations in physical activity, sexual dysfunction, and impaired body image, all of which can significantly reduce quality-of-life (QoL) (1–3). A normal aesthetic breast volume is generally considered to range between 200 and 400 mL (4–7). However, breast size varies considerably among populations and ethnic groups. According to data from the National Institutes of Health (NIH), the mean breast volume of Asian or Asian British women is approximately 454.8 mL, significantly lower than that of White (642.4 mL) or Black (777.8 mL) women (8). Similarly, a large prospective study of 605 Chinese women reported a mean breast volume of 340.0 ± 109.1 mL (9). These findings suggest that applying uniform volumetric or weight-based criteria derived from Western populations may be inappropriate for Asian women, underscoring the need for population-specific reference standards.

Previous studies have proposed anthropometric measurements and breast positioning relative to skeletal and soft tissue landmarks to guide aesthetic assessment and surgical planning (5, 10). The VECTRA® three-dimensional (3D) imaging system offers a non-invasive and reproducible method for assessing breast morphology, enabling accurate preoperative measurement of breast volume and related parameters without requiring surgical excision or tissue weighing (11).

The BREAST-Q questionnaire is a widely validated patient-reported outcome measure used to assess QoL across a range of breast conditions, including augmentation, oncologic reconstruction, and reduction mammaplasty. Evaluating QoL in Asian women with breast hypertrophy and identifying the anatomical and demographic factors that influence patient-reported outcomes are critical for optimizing surgical indication and patient counseling.

Although both 3D imaging and the BREAST-Q are well established, their combined preoperative application to generate a comprehensive morphological and QoL profile in Asian women with macromastia has not been adequately explored. Therefore, this study aimed to characterize preoperative 3D morphological features and patient-reported outcomes in Asian women with symptomatic macromastia using VECTRA® 3D imaging and the BREAST-Q, and to explore how objective anatomical parameters beyond breast volume relate to patient-reported outcomes. Ultimately, this work seeks to inform a more individualized and population-appropriate preoperative assessment framework.

## 2 Methods

### 2.1 Patients

Adult female patients were recruited from the Department of Plastic Surgery, The Second Affiliated Hospital, Zhejiang University School of Medicine, between June 2024 and May 2025. Based on commonly cited aesthetic reference ranges and previous studies, patients with a breast volume exceeding 400 mL were eligible for inclusion. All participants were evaluated preoperatively and had no history of prior breast surgery. Patients were assessed during surgical consultation for symptomatic breast hypertrophy, regardless of whether they ultimately proceeded to reduction mammaplasty. Demographic and clinical data were obtained from electronic medical records. Age groups were defined according to commonly used clinical age categories to facilitate exploratory comparisons of patient-reported outcomes. The studies involving humans were reviewed and approved by the Ethics Committee of the Second Affiliated Hospital of Zhejiang University School of Medicine (No. 2025-1448). The studies were conducted in accordance with the local legislation and institutional requirements. Written informed consent was obtained from the individuals for the participation in this study and publication of identifiable images and data included in this article.

### 2.2 Three-dimensional imaging

All patients underwent standardized 3D photography using the VECTRA® XT system (Canfield Scientific, USA). Automatically generated measurements included approximate breast volume, inter-breast volume difference, sternal notch-to-nipple (SNN) distance, clavicle-to-nipple distance, breast width, breast base width, nipple-to-inframammary fold (IMF) distance, areolar diameter, nipple-to-nipple distance, and intermammary distance. The degree of breast ptosis was assessed manually according to established criteria.

### 2.3 BREAST-Q questionnaire

Participants completed the Mandarin (CN) version of the BREAST-Q Version 2.0© Reduction/Mastopexy Module (Copyright © 2017, Memorial Sloan Kettering Cancer Center and the University of British Columbia). The questionnaire includes four preoperative scales: Psychosocial Well-being (9 items), Sexual Well-being (5 items), Physical Well-being (14 items), and Satisfaction with Breasts (11 items). Raw scores were converted to standardized scores ranging from 0 to 100 using the Q-Score program (https://webcore.mskcc.org/breastq/scoring.html), with higher scores indicating better outcomes.

### 2.4 Statistical analysis

Statistical analyses were performed using R software (version 4.4.2) and GraphPad Prism (version 10.2.0). Continuous variables were expressed as mean ± standard deviation (SD) or median with interquartile range (IQR), and categorical variables as frequencies and percentages. Group comparisons were conducted using independent t-tests or one-way analysis of variance (ANOVA) or Kruskal–Wallis tests for continuous variables and *χ*^2^ or Fisher's exact tests for categorical variables. Associations between continuous variables were evaluated using Pearson correlation analysis, whereas correlations involving ordinal variables were assessed using Spearman rank correlation coefficients. To examine the relationships between body mass index (BMI), breast morphological parameters, and BREAST-Q outcomes, univariable and multivariable linear regression analyses were performed. To account for potential nonlinear relationships between breast morphology and patient-reported outcomes, generalized additive models (GAMs) were applied. Statistical significance was defined as a two-tailed *P*value < 0.05.

## 3 Results

### 3.1 Patient demographics

A total of 145 patients underwent 3D imaging and 168 completed the BREAST-Q questionnaire. Fifty-three patients completed both assessments and constituted the final study cohort for analyses integrating morphological and QoL data. Demographic and breast morphological characteristics of the study cohort are summarized in Table 1. The median age was 34 years (range, 18–52 years), and the mean BMI was 24.1 ± 3.6 kg/m^2^. Nearly half of the patients were classified as young-middle-aged, and 58.5% had a normal BMI.

**Table 1:** Demographics and breast morphology of the study cohort.

Characteristic	Number	Mean ± SD	Median	IQR
Age (years)	53	34.2 ± 7.0	34	10.0
Young-adult (18–29)	15	26.2 ± 3.9	28	4.5
Young-middle-aged (30–39)	26	34.5 ± 3.2	34	5.8
Middle-aged (40–55)	12	43.2 ± 3.8	42	4.0
BMI (kg/m^2^)	53	24.1 ± 3.6	23.2	4.3
Underweight (< 18.5)	1	16.2		
Normal (18.5–23.9)	31	21.9 ± 1.4	22.4	1.7
Overweight (24.0–27.9)	12	25.9 ± 1.1	26.3	1.7
Obesity (≥ 28.0)	9	30.3 ± 1.5	30.1	2.3
Macromastia grade (mL)	53	773.0 ± 295.7	861.7	397.9
Mild (400 ≤ V < 600)	20	511.8 ± 56.5	511.5	66.0
Moderate (600 ≤ V < 800)	13	678.3 ± 49.6	675.3	38.5
Severe (800 ≤ V < 1,000)	9	910.0 ± 50.2	904.4	26.7
Gigantomastia (V ≥ 1,000)	11	1,235.8 ± 183.2	1,208.5	134.7
Mean Ptosis grade	53			
I	5			
II	35			
III	13			

V, volume; SD, standard deviation; IQR, interquartile range.

### 3.2 Three-dimensional imaging

**Figure 1:**
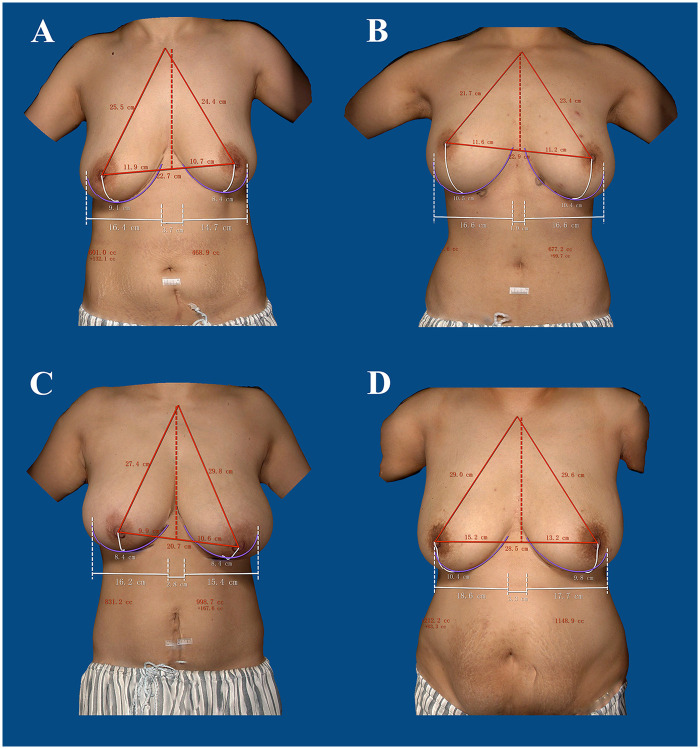
Classification of macromastia based on volumetric assessment. **(A)** Mild; **(B)** Moderate; **(C)** Severe; **(D)** Gigantomastia.

**Figure 2:**
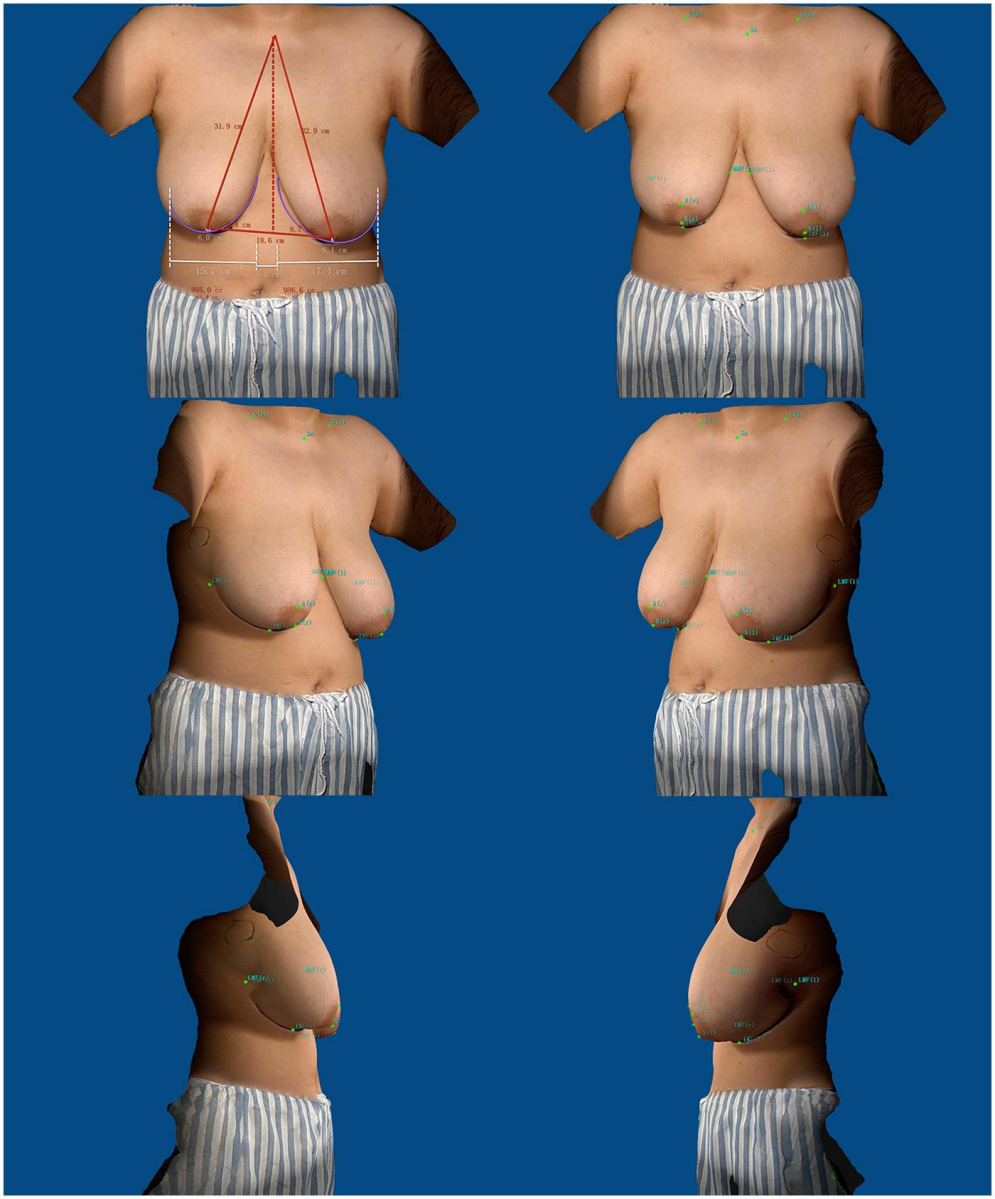
Three-dimensional photography of a patient with severe macromastia and grade III breast ptosis.

**Figure 3:**
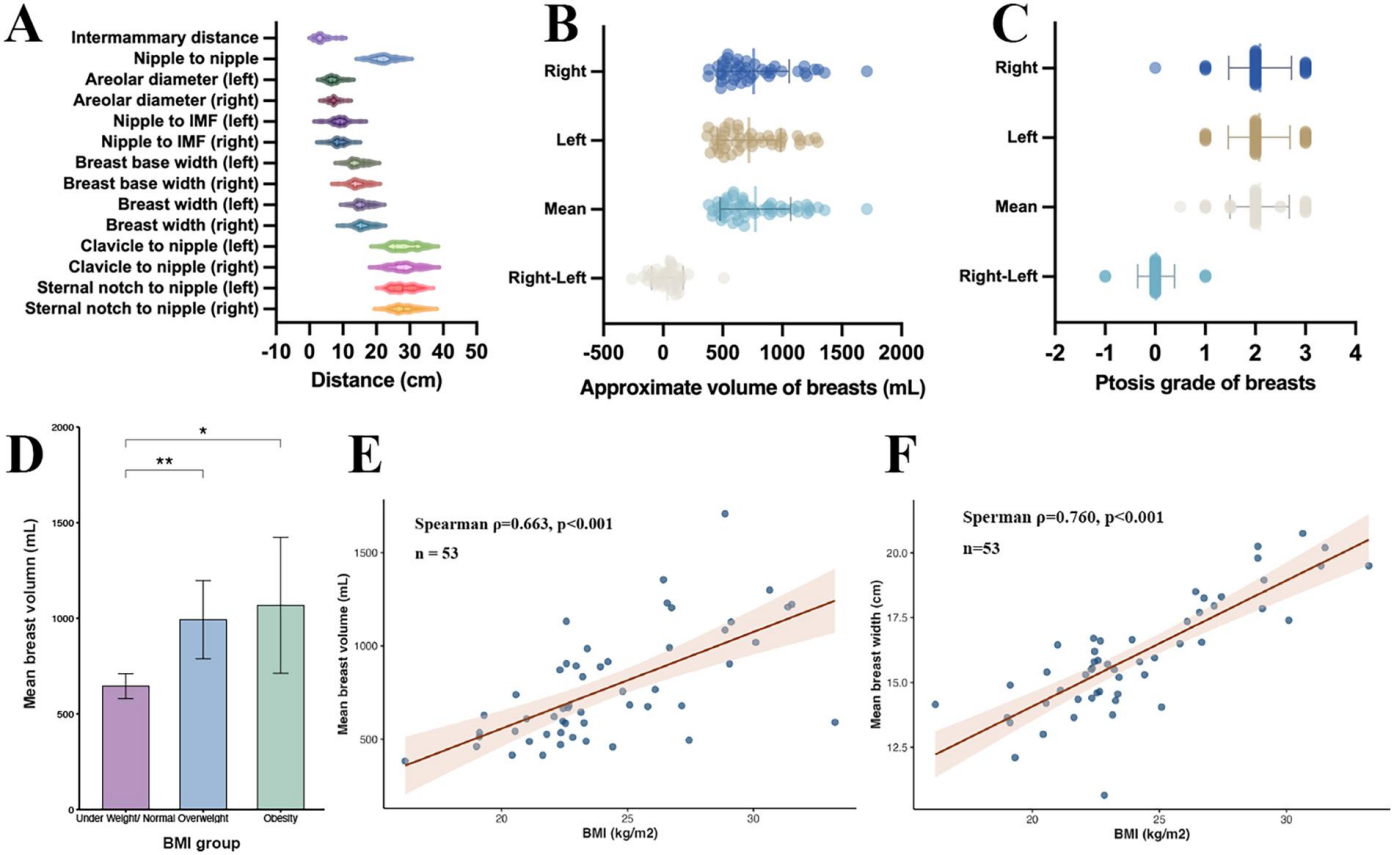
Quantitative breast morphological measurements and statistical analysis obtained using the VECTRA® XT 3D imaging system in 53 patients. **(A)** Landmark-based measurements of the breast; **(B)** Distribution of breast volumes; **(C)** Distribution of breast ptosis grades; **(D)** Comparison of mean breast volume across BMI groups by one-way ANOVA; **(E)** Association between BMI and mean breast volume, shown with a linear regression line and 95% confidence interval; **(F)** Association between BMI and mean breast width, shown with a linear regression line and 95% confidence interval. BMI: body mass index; Statistical significance: *P < 0.05, **P < 0.01, ***P < 0.001.

Comprehensive breast measurements were obtained using the VECTRA® XT system, with manual adjustment performed when necessary. Cases with incomplete reconstruction due to photographic artifacts were excluded. Macromastia was categorized according to volumetric assessment as mild (400–600 mL), moderate (600–800 mL), severe (800–1,000 mL), and gigantomastia (≥1,000 mL) (Figures 1, 2). Gigantomastia accounted for 20.7% of cases, whereas mild, moderate, and severe macromastia accounted for 34.0%, 24.5%, and 17.0%, respectively. Overall, 64.9% of individuals demonstrated right-breast volume dominance. Most patients with macromastia presented with grade II ptosis (66.0%), whereas grade I and grade III ptosis were observed in 9.4% and 24.5% of patients, respectively. Asymmetric ptosis was observed in 13.2% of the cohort (Table 1, Figures 3A–C).

BMI was positively correlated with mean breast volume (Pearson r = 0.642, *p* < 0.001; Spearman *ρ*= 0.663, *p* < 0.001) and mean breast width (Pearson r = 0.803, *p* < 0.001; Spearman *ρ*= 0.760, *p* < 0.001) (Figures 3D–F). Mean breast volume increased significantly across BMI categories, from 639.4 ± 184.7 mL in the underweight/normal BMI group to 851.0 ± 291.0 mL in the overweight group and 1,129.6 ± 303.0 mL in the obese group. Similarly, mean breast width increased from 14.9 ± 1.5 cm to 16.9 ± 1.4 cm and 19.4 ± 1.1 cm, respectively (Table 2). Significant differences in both breast volume and breast width were observed across BMI groups (ANOVA *p* < 0.001; Kruskal–Wallis *p* < 0.001 for both comparisons).

**Table 2:** Breast volume and width by Asian-adapted BMI group.

BMI group	*n*	Mean volume ± SD (mL)	Mean width ± SD (cm)
Underweight	1	382.9	14.2
Normal	31	647.9 ± 181.5	14.9 ± 1.5
Overweight	12	851.0 ± 291.0	16.9 ± 1.4
Obesity	9	1,129.6 ± 303.0	19.4 ± 1.1

BMI, body mass index; SD, standard deviation.

**Figure 4:**
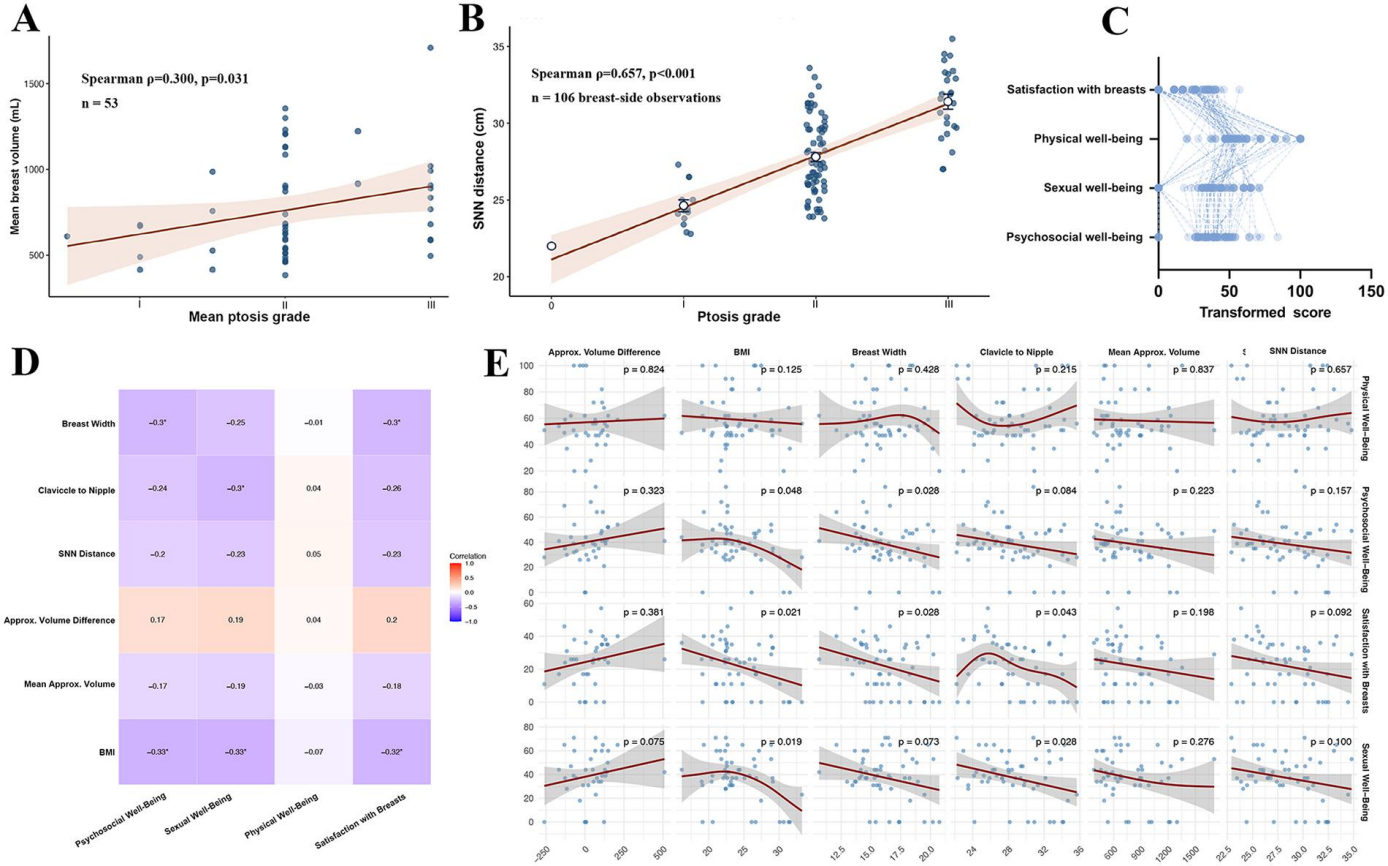
Correlation between breast morphology and BREAST-Q scales in 53 patients. **(A)** Association between mean ptosis grade and mean breast volume, shown with a linear regression line and 95% confidence interval; **(B)** Association between ptosis grade and SNN distance, shown with a linear regression line and 95% confidence interval; **(C)** Distribution of transformed BREAST-Q scores across the four domains; **(D)** Correlation matrix illustrating relationships among breast measurements, BMI and BREAST-Q domains; **(E)** GAMs demonstrating nonlinear associations between breast measurements and transformed BREAST-Q scores. SNN, Sternal notch-to-nipple; BMI, body mass index; GAMs, Generalized additive models; Statistical significance: *P < 0.05, **P < 0.01, ***P < 0.001.

Mean breast volume was positively correlated with ptosis severity (Spearman *ρ* = 0.300, *p* = 0.031), whereas SNN distance demonstrated a stronger positive correlation with ptosis grade (Spearman *ρ* = 0.657, *p* < 0.001) (Figures 4A,B). In the pooled 106 breast-side observations, SNN distance was significantly increased with clinical ptosis grade, supporting its use as an objective adjunct to clinical ptosis assessment (Table 3, Figure 4B).

**Table 3:** SNN distance according to ptosis grade for the right and left breasts.

Ptosis grade	*n*	Mean ± SD (cm)	Median (cm)
0	1	22.0	22.0
I	13	24.6 **±** 1.4	24.3
II	68	27.8 **±** 2.5	27.4
III	24	31.4 **±** 2.4	31.8

SNN, sternal notch-to-nipple; SD, standard deviation.

### 3.3 BREAST-Q questionnaire

**Figure 5:**
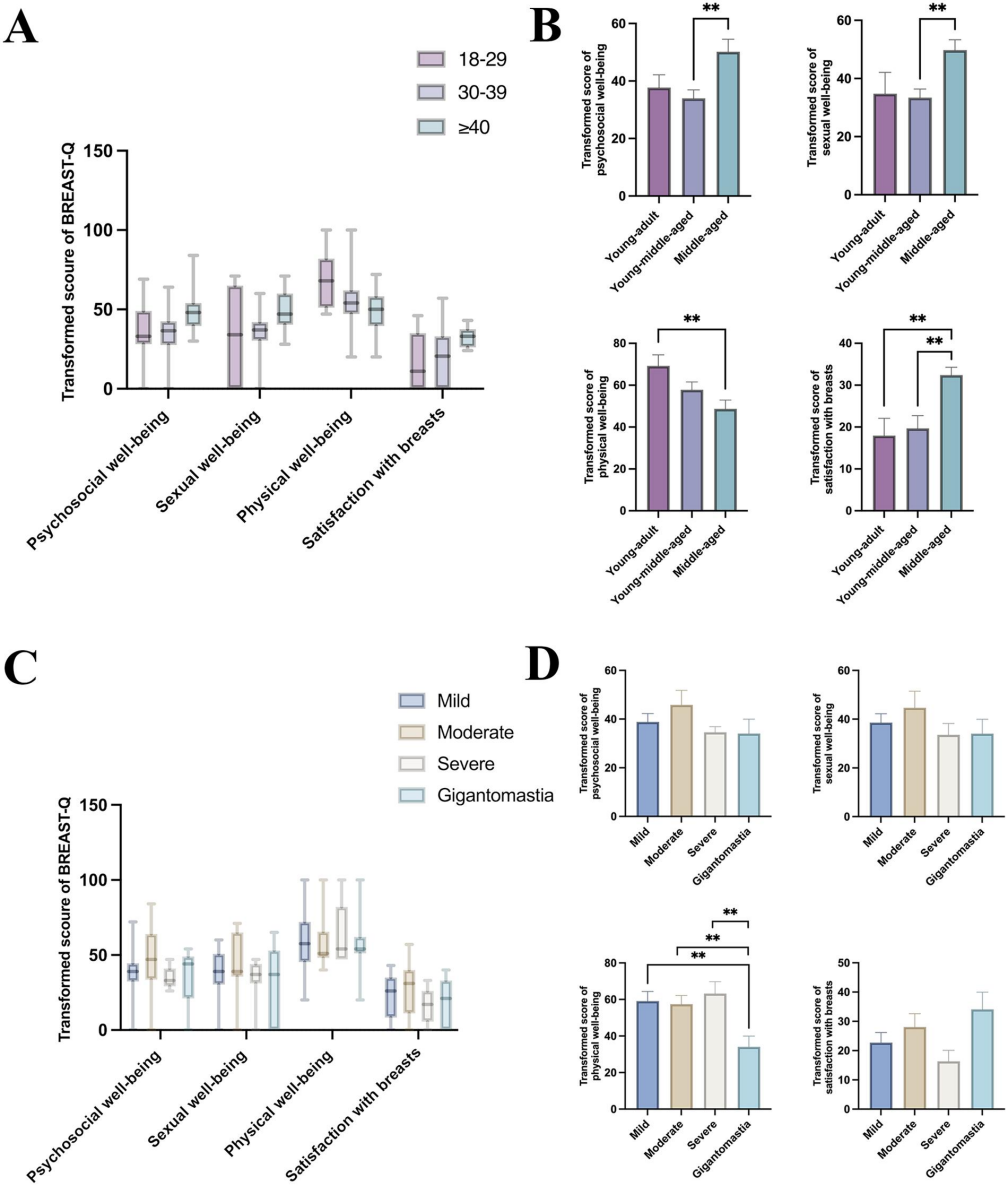
Associations of age and macromastia severity with BREAST-Q outcomes. **(A)** Box plots showing the distribution of transformed BREAST-Q scores across age groups; **(B)** Comparison of transformed BREAST-Q scores among age groups by one-way ANOVA; **(C)** Box plots showing the distribution of transformed BREAST-Q scores across volumetric macromastia categories; **(D)** Comparison of transformed BREAST-Q scores among volumetric macromastia categories by one-way ANOVA. Statistical significance: **P < 0.01.

Among BREAST-Q domains, satisfaction with breasts scores were lowest, followed by sexual well-being (Table 4, Figure 4C). All domains except physical well-being showed significant negative correlations with BMI (Figures 4D,E). Middle-aged patients reported higher scores across most domains, whereas physical well-being scores did not differ significantly among age groups (Figures 5A,B). Patients with gigantomastia reported significantly lower physical well-being scores than those with lesser degrees of macromastia (Figures 5C,D).

**Table 4:** Transformed BREAST-Q reduction/mastopexy module scores in patients with macromastia.

Scale	Mean ± SD	Median	IQR
Psychosocial well-being	38.7 ± 16.8	39	17
Sexual well-being	37.5 ± 20.0	37	19
Physical well-being	59.0 ± 19.7	54	21
Satisfaction with breasts	22.1 ± 15.0	26	24

SD, standard deviation; IQR, interquartile range.

Among all morphological and demographic variables, BMI demonstrated the strongest and most consistent negative association with psychosocial well-being, sexual well-being, and satisfaction with breasts. In linear regression analyses, BMI was independently associated with mean breast volume (*β*= 51.2 mL/kg/m^2^, 95% CI 33.4–69.1, *p* < 0.001) and mean breast width (*β*= 0.5 cm/kg/m^2^, 95% CI 0.4–0.6, *p* < 0.001). However, in multivariable models evaluating BREAST-Q outcomes, the associations of BMI, breast volume, and breast width with psychosocial well-being, physical well-being, sexual well-being, and satisfaction with breasts were attenuated and no longer statistically significant after adjustment for correlated variables (Table 5).

**Table 5:** Independent effects of BMI, volume, and width on BREAST-Q domains.

BREAST-Q outcome	Covariate	Estimate	*p* value	Independent effect
Psychosocial	BMI	−0.744	0.507	No
Psychosocial	Mean breast volume	0.004	0.746	No
Psychosocial	Mean breast width	1.780	0.408	No
Physical	BMI	0.356	0.781	No
Physical	Mean breast volume	−0.000	0.984	No
Physical	Mean breast width	−0.930	0.705	No
Sexual	BMI	−0.144	0.915	No
Sexual	Mean breast volume	−0.003	0.861	No
Sexual	Mean breast width	1.100	0.672	No
Satisfaction	BMI	−0.272	0.787	No
Satisfaction	Mean breast volume	0.010	0.386	No
Satisfaction	Mean breast width	−0.016	0.993	No

Statistical significance: *P* < 0.05.

## 4 Discussion

Macromastia is a prevalent condition associated with substantial physical and psychosocial burden. Although its precise etiology remains incompletely understood, hormonal influences, genetic predisposition, and obesity-related endocrine dysregulation are considered key contributing factors (12–15). Importantly, pronounced racial and ethnic differences in breast morphology have been consistently reported, challenging the applicability of a single universal definition of macromastia.

Historically, breast hypertrophy has been defined using a wide range of criteria, including breast volume, resection weight, and brassiere cup size (4, 16). Proposed thresholds have varied considerably, with breast volumes ranging from 350 to 800 mL and resection weights from 300 to 500 g used to define macromastia (7, 17). Gigantomastia has traditionally been defined as breast tissue exceeding 1,500 g per breast or accounting for more than 3% of total body weight (18). However, such definitions were largely derived from Western populations and surgical series, limiting their generalizability to Asian women. Breast density varies substantially between individuals and populations due to differences in adipose and glandular composition. Consequently, similar breast volumes may correspond to markedly different tissue weights, reducing the clinical relevance of weight-based thresholds, particularly for preoperative evaluation. Moreover, resection weight can only be determined intraoperatively, limiting its utility for surgical indication, patient counseling, and health system justification.

The VECTRA® 3D imaging system has demonstrated reliable accuracy in breast volume estimation, with reported errors of approximately 2%–5% compared with actual volume (19, 20). Previous studies have shown strong correlations between VECTRA-predicted and excised breast mass (21), supporting its role as a practical preoperative assessment tool. Although some studies have reported left-breast volume dominance (7, 22), our cohort demonstrated a predominance of right-sided volume, which may reflect population-specific differences or sample variability.

Consistent with prior literature, breast volume, ptosis severity, and SNN distance increased with BMI (23, 24). In our cohort, mean SNN distances exceeded traditionally cited aesthetic ideals, reflecting the strong association between macromastia and breast ptosis. Furthermore, the strong association between SNN distance and ptosis grade suggests that 3D imaging may facilitate objective characterization of breast ptosis and help distinguish true ptosis severity from apparent breast descent and serve as an objective adjunct to clinical assessment for surgical planning. Although pseudoptosis should still be determined by integrating nipple position, glandular descent, and the inframammary fold. Notably, 3D imaging facilitated more accurate identification of anatomical landmarks such as the IMF and lowest breast contour, which are often difficult to delineate using conventional photography in patients with large breasts.

Patient-reported outcomes highlighted the multifactorial nature of symptom burden in macromastia and suggested that absolute breast size may not fully capture patient distress. Although BMI and breast width were associated with several BREAST-Q domains in univariable analyses, these associations were not independently significant in multivariable models, likely reflecting the limited sample size and collinearity among anthropometric and morphological variables.

Importantly, 3D imaging revealed that breast volumes of approximately 1,000 mL or greater were associated with a disproportionate increase in ptosis severity and deterioration in physical well-being. These findings suggest that this volumetric threshold may represent a clinically relevant marker of increased functional impairment in Asian women with macromastia and warrants further investigation in larger cohorts.

Several limitations should be acknowledged. First, this was a single-center study with a relatively small sample size, which may limit the generalizability of the findings and preclude the establishment of definitive population reference standards. Second, the study population consisted of women seeking consultation for symptomatic macromastia at a tertiary plastic surgery center and may not be representative of the broader population. Third, information regarding comorbidities and ASA classification was not consistently available and therefore could not be incorporated into the analysis. Residual confounding by general health status cannot be excluded. Finally, the cross-sectional design precludes causal inference regarding the relationships between breast morphology, BMI, and patient-reported outcomes.

Based on these observations, breast volumes exceeding 1,000 mL may represent a clinically relevant threshold associated with increased ptosis severity and reduced physical well-being in Asian women with macromastia. Because this threshold was evaluated irrespective of BMI, it should be interpreted as a preliminary volumetric reference rather than a BMI-adjusted criterion. This proposal is not intended to replace existing weight-based definitions universally, but rather to complement them by providing a population-specific, preoperative framework that aligns with contemporary imaging techniques and better reflects patient burden.

By integrating objective 3D morphological assessment with validated patient-reported outcomes, this study provides preliminary preoperative morphological and QoL data for an underrepresented population and supports a multidimensional approach to surgical evaluation beyond reliance on breast volume alone.

## 5 Conclusion

Three-dimensional imaging and the BREAST-Q questionnaire offer complementary insights into the anatomical and psychosocial dimensions of macromastia. Our findings suggest that BMI and breast width were more strongly associated with patient-reported outcomes than breast volume in unadjusted analyses and should be considered alongside breast volume during preoperative assessment. Incorporating these readily obtainable parameters may facilitate more individualized surgical planning and improve patient counseling, particularly in Asian women with symptomatic macromastia.

## Data Availability

The raw data supporting the conclusions of this article will be made available by the authors, without undue reservation.
